# Walking recovers cartilage compressive strain in vivo

**DOI:** 10.1016/j.ocarto.2024.100526

**Published:** 2024-10-09

**Authors:** Shu-Jin Kust, Kyle D. Meadows, Dana Voinier, JiYeon A. Hong, Dawn M. Elliott, Daniel K. White, Axel C. Moore

**Affiliations:** a Department of Biomedical Engineering, University of Delaware, Newark, DE, USA; b Department of Physical Therapy, University of Delaware, Newark, DE, USA; c Department of Biomedical Engineering, Carnegie Mellon University, Pittsburgh, PA, USA

**Keywords:** Articular cartilage, Cartilage strain, Joint space width, Magnetic resonance imaging, Walking, Standing

## Abstract

**Background:**

Articular cartilage is a fiber reinforced hydrated solid that serves a largely mechanical role of supporting load and enabling low friction joint articulation. Daily activities that load cartilage, lead to fluid exudation and compressive axial strain. To date, the only mechanism shown to recover this cartilage strain in vivo is unloading (e.g., lying supine). Based on recent work in cartilage explants, we hypothesized that loaded joint activity (walking) would also be capable of strain recovery in cartilage.

**Methods:**

Eight asymptomatic young adults performed a fixed series of tasks, each of which was followed by magnetic resonance imaging to track changes in their knee cartilage thickness. The order of tasks was as follows: 1) stand for 30 ​min, 2) walk for 10 ​min, 3) stand for 30 ​min, and 4) lie supine for 50 ​min. The change in cartilage thickness was used to compute the axial cartilage strain.

**Results:**

Standing produced an average axial strain of −5.1 ​% (compressive) in the tibiofemoral knee cartilage, while lying supine led to strain recovery. In agreement with our hypothesis, walking also led to cartilage strain recovery. Interestingly, the recovery rate during walking (0.19 ​% strain/min) was nearly 3-fold faster than lying supine (0.07 ​% strain/min).

**Conclusions:**

This study represents the first in vivo demonstration that joint activity is capable of recovering compressive strain in cartilage. These findings indicate that joint activities such as walking may play a key role in maintaining and recovering cartilage strain, with implications for maintaining cartilage health and preventing or delaying cartilage degeneration.

## 1 Introduction

The fundamental role of articular cartilage is load bearing and smooth articulation of synovial joints. These biomechanical functions are driven by the pressurization of interstitial fluid during cartilage contact [[1], [2], [3]]. This pressurized fluid stiffens the cartilage, shields the solid matrix, and lubricates the sliding interface [4]. However, this same fluid pressure also drives fluid from the tissue, resulting in a loss of hydration [5] and fluid pressure, and increased compressive strain [6,7] and friction [6,8]. Maintaining and restoring cartilage hydration, and by effect, strain and friction are likely paramount to long-term cartilage function and health [9].

In vivo measures of human cartilage thickness, strain, and hydration are typically performed using magnetic resonance (MR) imaging, with a few studies also utilizing computed tomography [10], biplanar radiography [11,12], and ultrasound [13,14]. Studies have shown that static loading (e.g., standing) drives fluid from cartilage [[15], [16], [17]] with a consequential increase in cartilage compressive strain [11,16,18], which can surpass −16 ​% strain within 10 ​min [11]. On the other hand, static unloading (e.g., lying supine) restores cartilage thickness and hydration on the order of 0.05 to 0.09 ​% strain/min [19,20]. To date, the most studied in vivo loading environment is joint activity (knee bends [20,21], walking [[21], [22], [23]], running [21,24], cycling [21], single leg hops [25]), which produces cartilage strains on the order of −3 to −7.5 ​% [26]. This tissue strain can occur very rapidly, within 5 ​min of initiating activity [20,21,25], and remains nearly constant regardless of distance or duration (maximum distance studied to date has been 20 ​km with an estimated duration of 120 ​min) [24].

It is surprising that diurnal strains are only −5.1 ​% [27] when initial strain rates for static and active loading are on the order of −1 to −10 ​% strain/min [11,12,28], and static unloaded recovery rates are 0.08 ​% strain/min [20]. To achieve this level of diurnal strain would require a person to unload their joints for much longer than they load them. However, according to the US Bureau of Labor Statistics (2022), the median civilian worker spends more than 50 ​% of their day loading their joints (standing, walking, or climbing). Given this, it seems likely that another mechanism of cartilage recovery must exist.

In the 1960's, Linn observed cartilage strain recovery in excised dog ankles slid against a metal bearing surface [29]. It was proposed that recovery occurred due to unloading and exposure of the cartilage contact on each cycle. In 2016 this was revisited by Moore and Burris who showed that sliding cartilage without contact exposure or unloading could also yield strain recovery [30,31]. Since both contact exposure, unloading, and sliding will occur during joint articulation it is possible that joint activity is capable of cartilage strain recovery.

In this study we aim to determine if in vivo joint activity (i.e., walking) can recover cartilage strain or only maintain strain. Our hypothesis is that in vivo joint activity is capable of cartilage strain recovery. If true, this hypothesis provides a mechanism by which large cartilage strains, such as those produced during prolonged standing, can be recovered, and how diurnal strains are maintained throughout a day. Our secondary objective is to compare the recovery rates of joint activity to static unloading, the only other known in vivo recovery mode.

## 2 Methods

Following Institutional Review Board approval, we recruited 8 human participants with asymptomatic knees. Participants were 23 to 35 years of age (29 ​± ​3 years, mean ​± ​95 ​% confidence interval). This narrow age range was used to control for potential age-related changes to articular cartilage [[32], [33], [34]]. Eligibility was determined through a pre-screening questionnaire and excluded participants with prior knee injury. During the study, participants also completed a Knee Injury and Osteoarthritis Outcome Score (KOOS) questionnaire for pain and symptoms. The KOOS is a semi-quantitative tool to measure patient reported outcomes that relate to knee osteoarthritis [35]. A KOOS of 0 ​= ​extreme pain and dysfunction, while 100 ​= ​no pain or impact on daily life.

Following eligibility screening, participants were given a wrist worn activity tracker (Pro-Fit Active, Octandra) that recorded the number of steps taken over a minimum of 24 ​h. If the activity tracker was worn for more than 24 ​h then the average 24 ​h step count is reported.

### 2.1 Participant characteristics

Participant characteristics are listed in Table 1. A power analysis identified a study group size of N ​≥ ​7 (G∗Power 3.1, α ​= ​0.05, β ​= ​0.8, change to detect ​= ​1 ​%, standard deviation ​= ​0.7 ​%). While we maintained balanced biological sex (4 male and 4 female), we did not aim to detect significant effects of sex; thus, our sample sizes are underpowered for this type of analysis.

**Table 1:** The mean, range, and ±95 ​% confidence interval for participant characteristics and secondary measures. Participant age, BMI, and daily steps are similar to previous work investigating young asymptomatic adults [36,37]. Note that stance angle was recorded for seven of the eight participants. All participants were right hand dominant.

Parameter	Mean	Range	±95 ​% Confidence Interval
Age (years)	29	23 to 35	3
BMI	23	19 to 26	2
Dominant Hand	Right	–	–
Daily Steps (1000's)	13	4 to 25	5
KOOS – Pain	100	97 to 100	1
KOOS – Symptoms	96	82 to 100	4
Stance Angle (deg), extension is negative	−2	−16 to 10	6
Distance Walked in 10 ​min (m)	750	590 to 940	100
Walking Speed (m/s)	1.3	0.8 to 1.6	0.2

### 2.2 Task Protocols

**Fig. 1:**
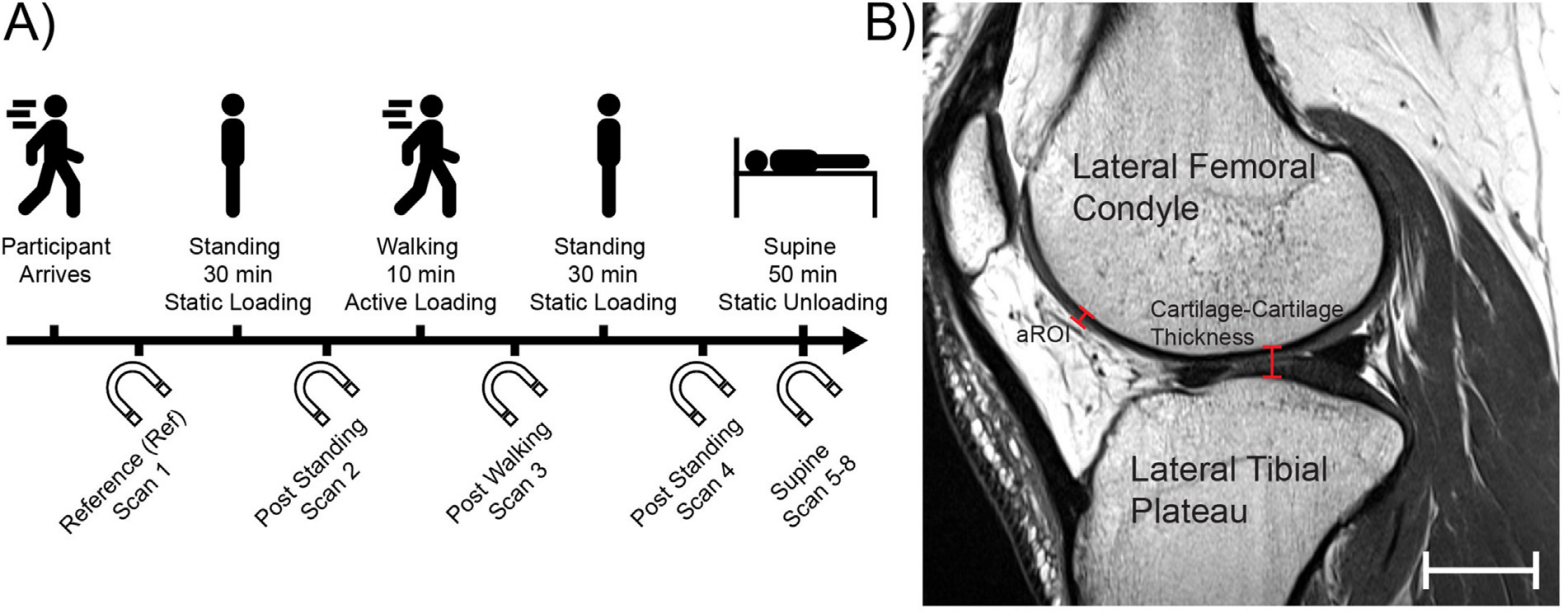
A) Task and MR scanning protocol. The sequence of events proceeds from left to right. B) Sagittal view of a representative knee joint. A line is shown along the superior-inferior axis in the central region of cartilage-cartilage contact in the lateral tibiofemoral compartment to demonstrate the method of measuring the bone-to-bone distance (cartilage-cartilage thickness). An anterior region of interest (aROI) is shown on the anterior surface of the lateral femoral condyle (thickness is measured normal to the underlying bone). All thickness measures are performed in the sagittal plane which has an in-plane image dimension of 0.34 x 0.34 ​mm. Scale bar ​= ​20 ​mm (bottom right corner).

On the day of MR scanning, the participant arrived at the MR facility and underwent an MR safety assessment before beginning the study. A baseline reference (Ref) scan, see Section 2.3 *MR Scan Sequence,* was taken of their right knee. The participant was removed from the scanner and completed the remaining fixed order of tasks, each of which was followed by MR imaging: stand for 30 ​min (static loading), walk for 10 ​min at 110 steps per min (active loading), stand for 30 ​min, and remain supine for 50 ​min (static unloading). During the final task, lying supine, the participant was scanned 4 times at equally space intervals (∼13 ​min). Additional details for static loading, active loading, and static unloading are described below and in Fig. 1A.

#### 2.2.1 Static loading

Participants were asked to stand upright in a comfortable position and avoid locking out their knees. During prolonged standing it is natural for participants to weight shift and fidget [38]. We found that securing a taut Kimwipe (11 ​× ​21 ​cm sheet) over the anterior side of each knee provided a slight physical cue that reduced joint motion and did not over constrain the joint or cause an unnatural stance. If the participant flexed their knee during the standing task, the Kimwipe was pulled into tension, and if pulled far enough it would break. We only recorded one break for the entire cohort. For this participant, the Kimwipe was promptly replaced, and the study continued. During the standing task, with the Kimwipe secured, we measured the stance angle between the greater trochanter (hip), lateral epicondyle (knee), and lateral malleolus (ankle). We identified and marked these anatomical locations by palpation, took a digital photograph, and measured the stance angle in ImageJ, see Table 1. Following static loading (30 ​min), participants were asked to walk stiff legged (not bending at the knee) into the MR room, a distance of 7 ​m, and then lay on the MR bed while the knee coil was positioned. We did not record any breaks during this short excursion; however, most participants broke the Kimwipe when lifting their legs onto the MR scanner bed.

#### 2.2.2 Active loading

Following the first static loading period, the participants were scanned and then walked 7 ​m out to a straight hallway. The participants were asked to walk continuously up and down the hallway (16 ​m long) for 10 ​min at 110 steps per minute (a typical cadence for this age group [39]). The pace was set by a digital metronome and the walk duration was timed by a digital timer that started once the participant began walking from the MR scanner. The total distance walked was measured via a distance wheel, see Table 1. Participants walked at an average speed of 1.3 ​m/s, which is typical given the participant characteristics [40]. Following this, participants returned to the MR scanner and were immediately positioned and scanned. The time (mean ​± ​standard deviation) to remove the participant from the scanner, perform the 10 ​min walk, reposition (physical positioning and adjusting scan location), and start data collection was 12 ​min 45 ​s ​± ​1 ​min 9 ​s.

#### 2.2.3 Static unloading

Following the second static loading period the participants were positioned in the MR scanner and remained in a supine position for the duration of the static unloading period. Scans occurred approximately every 13 ​min. The mean duration of static unloading was 51 ​min 26 ​s.

### 2.3 MR Scan Sequence

A 3T Siemens PRISMA scanner equipped with a 15 channel Tx/Rx knee coil was used to scan the right knee of participants in the sagittal plane (Fig. 1B) using a Proton Density weighted Turbo Spin Echo (PD-TSE) sequence (TR ​= ​9710 ​ms, TE ​= ​37 ​ms, Flip Angle ​= ​150 deg). The field of view was 13 ​× ​13 ​cm [384 x 384 px] with a slice thickness of 1.5 ​mm. The resulting voxel size was 0.34 x 0.34 ​× ​1.5 ​mm. The scan duration was 3:43 ​min.

### 2.4 MR image analysis

MR image analysis was performed in 3D Slicer [41], an open-source image analysis software. PD-TSE scans (full joint) for each participant were registered using a 6 degree of freedom rigid transformation to the Ref scan. The central region of cartilage contact for each compartment (lateral and medial) were identified on the Ref scan. The distance from the bone-cartilage interface of the femur to the bone-cartilage interface of the tibia was measured in the central region of contact along the superior-inferior axis for each compartment using a 1 px wide line, see Fig. 1B. The line location was then mapped on to all subsequent scans which were registered to the Ref scan. We updated the line length to account for changes in the bone-to-bone distance (tibiofemoral cartilage thickness). The change in the bone-to-bone distance relative to the Ref scan (*L*_*Ref*_) was used to calculate the axial cartilage strain (engineering strain): $Strain\;\left(\%\right)=\frac{L_{i}-L_{Ref}}{L_{Ref}}\cdot 100\%$. Note that axial strain is the only component of strain measured and reported in this work. We use the terms axial and compressive strain throughout to explicitly indicate this.

Intra-rater repeatability was performed on all scans, in each compartment, for all participants. A total of 128 (8 participants, 8 scans per participant, 2 knee joint compartments) repeated measures were made without reference to the original measurement location. Intra-rater repeatability bias, standard deviation, and ±95 ​% confidence interval were −0.2, 0.7, 0.1 ​% strain, which is comparable with other cartilage MRI studies [42,43]. See Fig. S1 for a graphical representation of this data.

Inter-rater repeatability was performed between 2 raters on all scans, in each compartment, for all participants. A total of 128 (8 participants, 8 scans per participant, 2 knee joint compartments) repeated measures were made without reference to the other rater's measurement location. Inter-rater repeatability bias, standard deviation, and ±95 ​% confidence interval were −0.3, 0.9, 0.2 ​% strain, see Fig. S2 for a graphical representation of this data. All measures reported in the results are from rater 1.

A second region of interest was chosen on the anterior surface (aROI) of the lateral femoral condyle for each participant, Fig. 1B. This location is not in cartilage-cartilage contact while the participant is supine, and we do not expect contact to occur during the standing task; however, there may be contact during the walking task depending on the degree of knee flexion. Therefore, we hypothesized that the strain in the anterior region of interest would be negligible with the exception of the walking task.

### 2.5 Statistics

Unless noted otherwise, we report the mean ​± ​95 ​% confidence interval for all 8 participants in this study. We evaluated normality using Shapiro-Wilk's test (Origin 2022) and variance using Levene's test (JMP Pro 17). Normality was not met across all conditions; therefore, we used Kruskal-Wallis to detect significant effects.

If a significant effect was detected, we used Dunnett's Method to identify significant differences. All post-hoc statistical tests are with respect to the first static loading period to limit our findings to the most relevant based on our questions: 1) does active loading produce significant compressive strain recovery, 2) does subsequent static loading produce a significantly different compressive strain, and 3) confirm static unloading produces significant compressive strain recovery. Significance was set at p ​< ​0.05 for all statistical tests.

## 3 Results

**Fig. 2:**
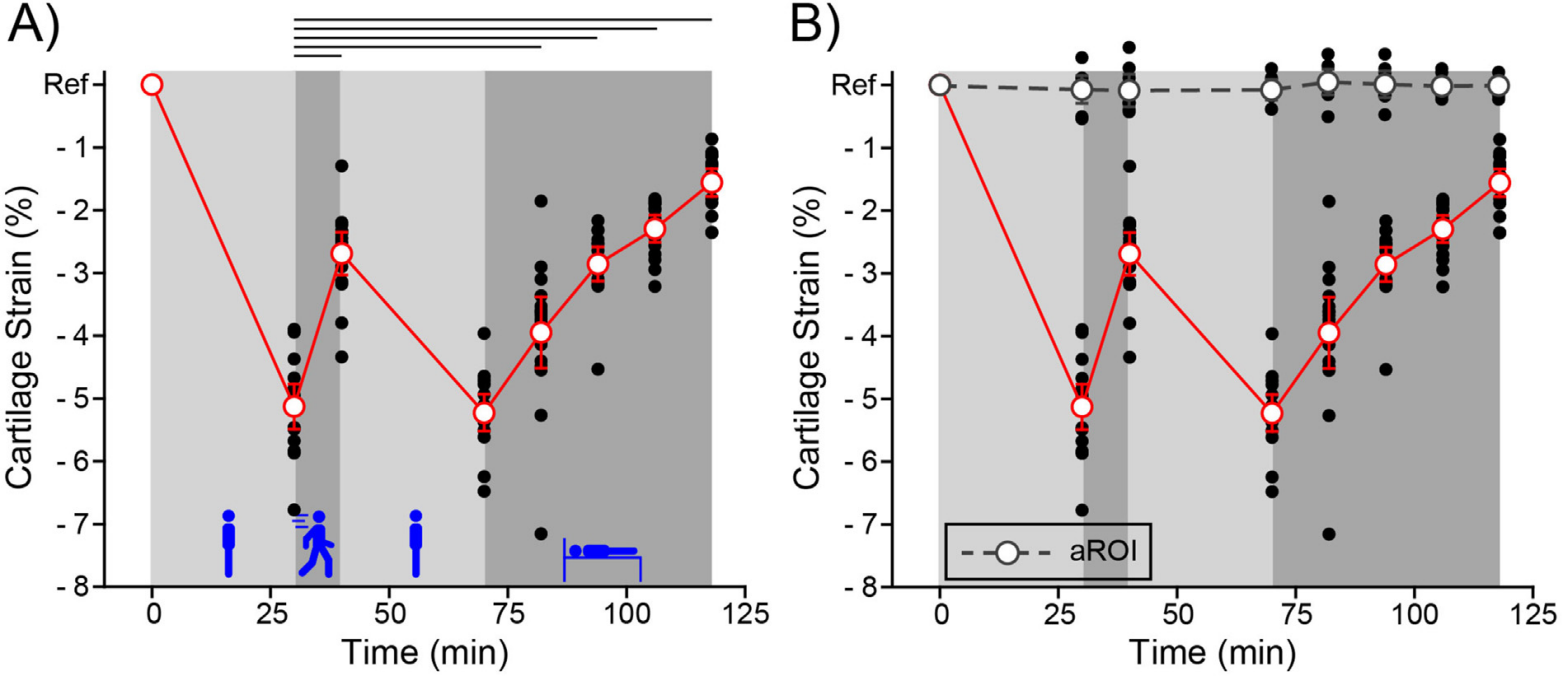
Cartilage strain relative to Ref following each activity phase (8 timepoints, 8 participants, 2 compartments measured per participant). Individual participant measures (black dots) and the mean ​± ​95 ​% confidence interval are shown (open circles and error bars). A) Cartilage strain was compressive in the central region of contact (red circles and red line) in the medial and lateral tibiofemoral compartments. Activity phase was found to have a significant effect on strain (p ​< ​0.001). Lines above the plot indicate significantly different pairs. B) Cartilage strain in the aROI (grey circles and dashed line). The effect of task on the axial strain in the aROI was not significant (p ​< ​0.91). Data from the region of contact (red circles and red line) is from Fig. 2A. Note that we define Ref strain; therefore, we do not include it when performing statistical tests.

The combined axial cartilage strain in the lateral and medial compartments is shown in Fig. 2A. We detected a significant effect (p ​< ​0.001) of task on cartilage strain. All tasks produced a significantly different strain when compared to the first static loading (standing) period except for the second static loading period.

Static loading (standing for 30 ​min) produced a residual axial cartilage strain (−5.1 ​± ​0.4 ​% strain), while static unloading (lying supine for 51 ​min) led to monotonic strain recovery (3.7 ​± ​0.2 ​% strain recovery). In agreement with our hypothesis, active loading (walking for 10 ​min) led to strain recovery (2.4 ​± ​0.2 ​% strain recovery). We term this novel activity driven recovery mode *Active Recovery* to distinguish it from *Passive Recovery* that occurs during static unloading.

In addition, we quantified the axial strain in the anterior region of interest shown in Fig. 1B. The cartilage strain in this region is negligible (−0.02 ​± ​0.18 ​% strain) and was not significantly affected by task (p ​< ​0.91), see Fig. 2B. The lack of cartilage strain and relatively small variance in this region suggests that contact did not occur in this location during the walking task; however this remains to be proven.

**Fig. 3:**
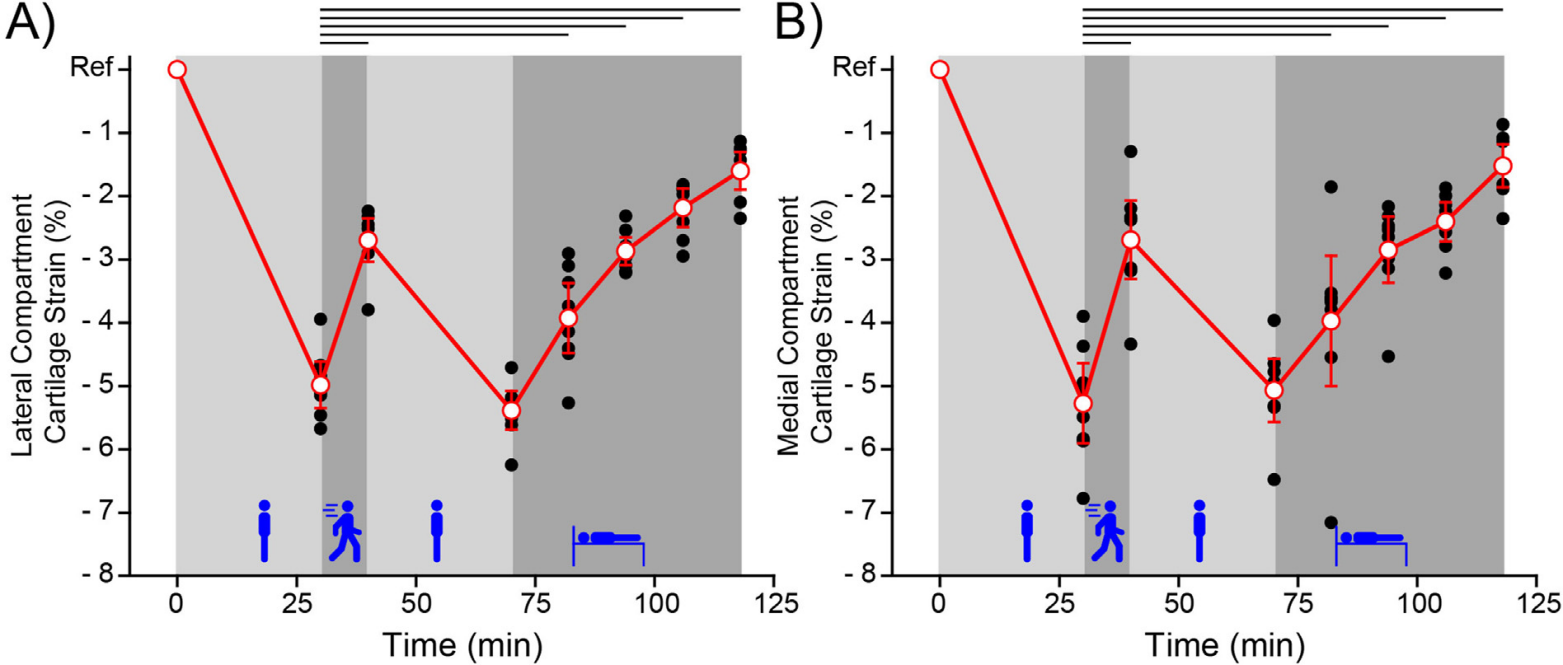
A) Lateral and B) Medial compartment axial strain relative to Ref following each task. Individual participant measures (black dots) and the mean ​± ​95 ​% confidence interval are shown (red circles and error bars). Task was found to have a significant effect on strain in both the lateral (p ​< ​0.001) and medial (p ​< ​0.001) compartments. Lines above the plots indicate significantly different means. With respect to the first static loading period, every subsequent timepoint was significantly different except for the second static loading period. Participant specific data is shown in Fig. S3. Note that we define Ref strain; therefore, we do not include it when performing statistical tests.

Individual compartmental (lateral and medial) cartilage strain is shown in Figs 3A and B, and is nearly identical. Task had a significant effect (p ​< ​0.001) on lateral and medial compartmental cartilage strain. All tasks produced significant changes in compartmental strain when compared to the first static loading (standing) period except for the second static loading period. In the following sections we perform a more detailed analysis of each activity phase.

### 3.1 Static loading

**Fig. 4:**
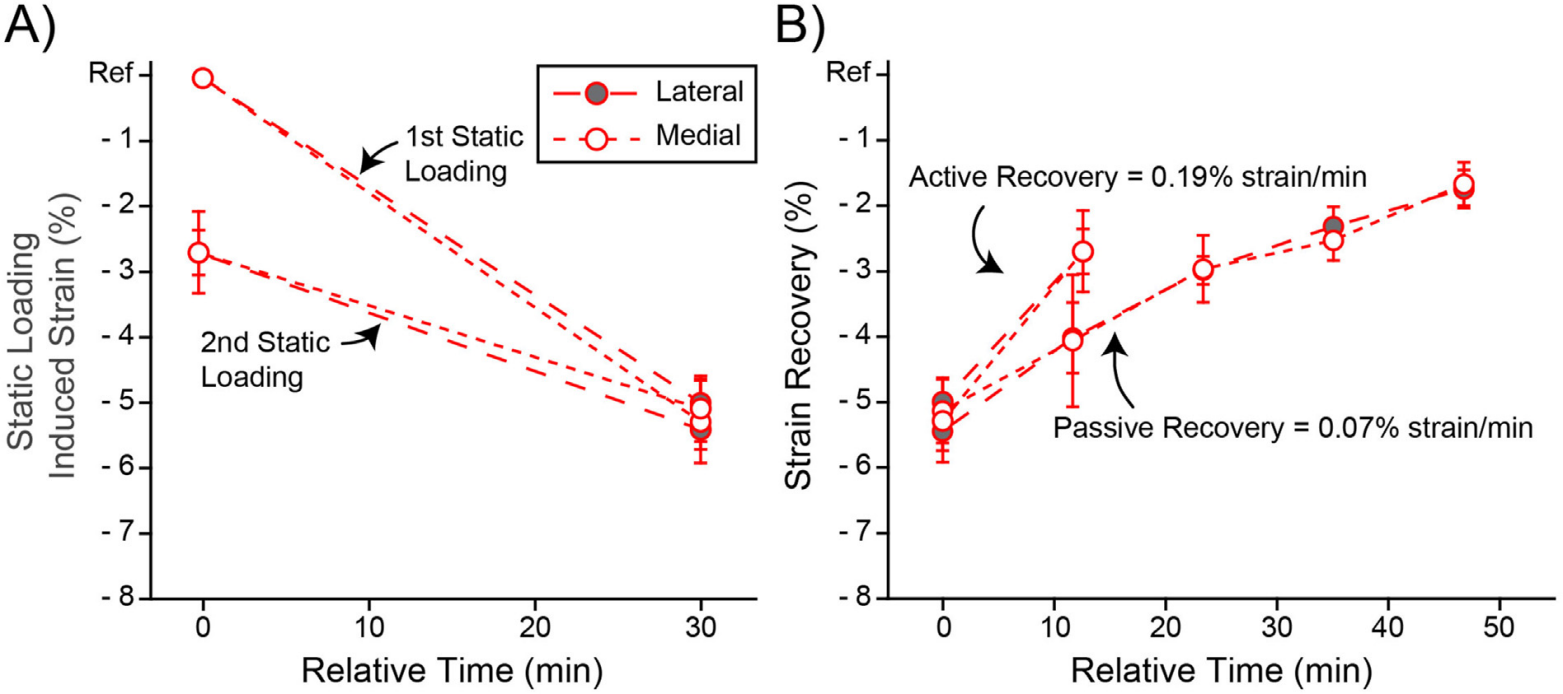
A) First versus second static loading period for both lateral and medial compartments. Notice that the compressive strain after 30 ​min is similar between the first and second loading period despite starting from different initial compressive strains. There was no significant effect of compartment (lateral versus medial, p ​< ​0.49) nor the strain after 30 ​min of static loading (first versus second static loading, p ​< ​0.17). B) Active and passive strain recovery for both lateral and medial compartments. Active recovery was not significantly different between the two compartments (p ​< ​0.96). Passive recovery was not significantly different between the two compartments at any timepoint (p ​< ​0.96, 0.43, 0.23, and 0.79). Average active and passive recovery rates are shown. Note that our calculation of the active recovery rate includes the subject positioning time (2:45 ​min) in addition to the walking duration (10 ​min). This gives a conservative estimate of the active recovery rate.

Static loading produced an expected compressive strain response. We observed a −5 ± 0.4 ​% and −5.3 ​± ​0.6 ​% strain in the lateral and medial compartments during the first static loading period and −5.4 ​± ​0.3 ​% and −5.1 ​± ​0.5 ​% strain during the second static loading period, Fig. 4A. The effect of compartment (lateral versus medial) was not significant, p ​< ​0.49. Furthermore, the strain after the first and second static loading period was not significantly different, p ​< ​0.17.

### 3.2 Recovery

Both active loading and static unloading produce compressive strain recovery in articular cartilage as shown in Fig. 3. Below we analyze each of these recovery modes.

Active loading (walking for 10 ​min at 110 steps per min) reduced the compartmental strain to −2.7 ​± ​0.3 ​% and −2.7 ​± ​0.6 ​% in the lateral and medial compartments respectively. Compartmental strain was significantly recovered from the preceding static loading strain but was not significantly different between the two compartments (p ​< ​0.96), see Fig. 4B. In other words, the lateral and medial compartments started at similar strains and after walking for 10 ​min they recovered a significant amount of strain but were indistinguishable from one another. If we assume that the rate of recovery is linear, then active loading (walking) recovers 0.19 ​% strain/min for the articular cartilage of the knee. Note that this calculation includes the 10 ​min walk in addition to the subject positioning time (2:45 ​min) to give a conservative estimate of the recovery rate. While this recovery rate is a useful guideline, it is unlikely that active recovery is linear over the walk duration.

Passive recovery (lying supine for 51 ​min) significantly reduced the compartmental strain to −1.6 ​± ​0.3 ​% and −1.5 ​± ​0.3 ​% in the lateral and medial compartments. Compartmental strain was not significantly different between the two compartments at any timepoint (p ​< ​0.96, 0.43, 0.23, 0.79), see Fig. 4B. In other words, the lateral and medial compartment started at similar strains, static unloading produced significant recovery in both compartments, and the recovery was similar for both compartments. Assuming that the rate of recovery is linear, which the data generally demonstrate, then passive recovery yields a 0.07 ​% strain/min recovery rate. This passive recovery rate is in excellent agreement with the work of Eckstein et al. [20].

## 4 Discussion

The central hypothesis of this study was that in vivo joint activity is capable of compressive strain recovery in articular cartilage. Our objectives were: (1) demonstrate active recovery and (2) compare active and passive recovery rates.

Addressing our central hypothesis and objective (1), we have shown for the first time that significant cartilage strain recovery in vivo is possible through walking (active loading), see Fig. 2, Fig. 3, Fig. 4. We term this novel in vivo recovery mode *Active Recovery*. Prior to this work, the only other known in vivo cartilage recovery mode was passive recovery (static unloading). While the physiological importance of active recovery has yet to be established, we hypothesize that it is a primary regulator of cartilage strain during waking hours and is the primary reason our diurnal strains are only −5 % [27].

It may be interesting that despite numerous in vivo studies of activity and cartilage strain [26] that this is the first study to document activity induced cartilage strain recovery. To date, all other studies that have investigated the effect of activity have shown that the residual compressive strain increases up to ​∼ ​−5 % strain, after which it is maintained at this level of strain [20,22,26,36]. We were able to observe cartilage recovery because our protocol was designed to detect it by first establishing a residual compressive strain, via standing, prior to the walking activity.

Having identified a new mode of recovery, it is worth asking which mode of cartilage recovery is faster, objective (2). Based on the recent work of Voinier et al. we hypothesized that active recovery would be faster than passive recovery [31]. We performed linear regressions on each participant's recovery data to calculate a recovery rate. Comparing the active and passive recovery rates, we find a significant difference (p ​< ​0.0001) in which active recovery is almost 3-fold faster (Fig. 4B), which is in direct agreement with the work of Voinier et al. and our hypothesis. Initially, this seems to indicate active loading is a superior recovery mode. However, we do not yet know what effect activity duration has on active recovery. Longer periods of activity may yield diminishing returns and become less competitive [31]. A possible advantage of passive recovery is that it is capable of more complete cartilage recovery as it minimizes the load on the joint, which is the driving force for fluid exudation and cartilage strain [20]. Finally, the differences in recovery rate indicate that the underlying mechanism of active recovery is fundamentally different from passive recovery. In future work we will elucidate the mechanics that drive active recovery. Potential mechanisms may include passive recovery due to periods of unloading [20], free swelling due to cartilage unloading and contact exposure [31], mechanical pumping due to cyclic loading [3], and sliding induced fluid recovery due to the relative tangential motion of the contacting surfaces [30].

An unexpected but interesting finding from this study was the compressive strain after the first (−5.2 ​% strain) and second (−5.3 ​% strain) static loading periods (Fig. 4A) were not significantly different. We anticipated that the second static loading period would lead to a greater strain than the first since there was an average residual strain of −2.7 ​% after 10 ​min of active recovery. Based on other published work, it is highly unlikely that −5 % strain is the limit of in vivo cartilage deformation [10,11,43,44].

Several factors may contribute to this discrepancy. First, participants lie supine during imaging. This effectively unloads their cartilage during scanning and allows stored elastic stress in the cartilage to recover [43]. Second, in our study static loading is performed in a two-legged stance with roughly equal weight distribution (50 ​% body weight per leg), while Uzuner et al. applied approximately 50 ​% body weight in a single leg stance [11]. Furthermore, Herberhold et al. loaded cadaveric knee joints to 150 ​% body weight at 60 deg of flexion [44]. While these and other factors may explain some of the discrepancy, we propose that an additional, and potentially more significant, effect is motion (fidgeting and weight shifting) during standing [38,45]. While we aimed to minimize participant movement through verbal and slight physical cues (Section 2.2 *Task Protocols*), we did not want to create an abnormal or unsafe (e.g., locking out knees) loading environment. To quantify this motion (fidgeting and weight shifting) during stance, we recruited 1 participant from the study and had them perform the same 30 ​min standing task as described in Section 2.2 *Task Protocols*. The participant wore reflective markers on their tibia and femur. These bodies were tracked using four OptiTrack Prime 13 cameras at 100 ​Hz. Over 30 ​min of standing, the mean absolute value for range of motion (flexion-extension) and angular velocity were 0.79 deg and 0.74 deg/s. While these are small rotations and angular speeds, they are none the less a motion, which has been shown to limit strain [22,46].

### 4.1 Advantages and limitations of the study design

There are many advantages of this study design; however, it is not without its limitations. Below we discuss some of the key advantages and limitations of our approach: (1) small sample population, (2) MR sequence, (3) pre-study protocol and reference state, (4) measurement technique.

Our primary goal was to test the hypothesis that activity (walking) is a recovery mechanism. Based on a power analysis from pilot data and prior work in the field [23,25] we determined that 8 participants was a sufficient sample size to demonstrate this phenomenon in a young and asymptomatic cohort. Our narrow recruitment characteristics likely helped to maximize our observation of active recovery by minimizing factors such as age-related changes to cartilage.

The use of PD-TSE scans is a deviation from current cartilage research practice that more commonly implements DESS [34,47] or SPGR sequences [18,36,43] due to higher signal to noise [48]. We choose to use proton density weighting as it is a routine clinical scan used in assessing knee pathology, allows for good scan resolution, and provides contrast between cartilage, bone, meniscus, and fluid [37,49]. Furthermore, the PD-TSE only takes 3:43 ​min, which minimizes the effects of unloaded recovery while scanning.

Our pre-study protocol deviates from prior work in some important ways. First, we did not restrict participant activity (lifting, running, etc.) on the day of, or the day prior to, MR scanning. Second, we did not control the time of day in which MR scanning took place. Finally, we did not unload participants (lying supine or sitting) prior to their Ref scan. Restricting activity [22,50], scanning just after waking (e.g., 7:00 a.m.) [22,50], and placing participants in an unloaded state [17,21,25] aim to maximize cartilage hydration and minimize the residual compressive strain [20]. It should be noted that it is unlikely that ‘unloading’ a joint in vivo produces zero load on the contacting cartilage surfaces. Therefore, even extensive periods of static unloading are unlikely to yield 0 ​% strain. In this study, we aimed to observe changes in cartilage strain relative to each participant's daily norm; thus, we define the first MR scan as our reference (Ref) scan. All measures are made relative to Ref. Prior work has shown that this Ref strain is on the order of −5 % and can be achieved in as little as 5 ​min of active loading [20,21,25]. The advantage of our approach is that it evaluates cartilage dynamics under typical waking hour conditions (non-zero strain) and demonstrates methodological robustness (i.e., a strict pre-study protocol is not necessary to detect the effects reported in this work).

An important advantage of our method for measuring cartilage strain is its potential for translation. Measuring changes in the tibiofemoral cartilage thickness at single location in each compartment is fast and simple when compared to manual cartilage segmentations. Furthermore, this method is similar in principle to measuring joint space width, a clinical tool for classifying osteoarthritis severity. The major limitation of our method is its susceptibility to measurement error. Using our method, if the user incorrectly identifies the cartilage-bone interface it can greatly skew the results. Alternatively, when segmentations are performed, regional thickness measures are often averaged together [47], which can dampen the effects of a few poorly chosen interfaces. Despite the potential limitation of our method, we achieved an intra-rater repeatability (standard deviation of the difference) of ±0.7 ​% strain, inter-rater repeatability (standard deviation of the difference) of ±0.9 ​% strain, and the necessary sensitivity to conclusively answer our study questions.

## 5 Conclusion

In this work we report the first demonstration that activity (walking) is an in vivo mechanism of compressive strain recovery in articular cartilage which confirms our primary hypothesis. Our secondary objective was to compare the rates of active and passive recovery. We observed that active recovery is more than 3-fold faster than passive recovery, the only other known mechanism of in vivo cartilage recovery. This first demonstration of active recovery in vivo lays the groundwork for future studies that investigate the effects of activity on cartilage.

## Author contributions

All authors contributed significantly to this work: project design (DV, DME, DW, ACM), data collection and analysis (SJK, KDM, JAH, ACM), writing (SJK, KDM, DV, JAH, DME, DW, ACM), and funding (DME, ACM).

## Declaration of competing interest

The authors have no competing interests to declare.
