# Supplementary material for: Walking recovers cartilage compressive strain in vivo
Source: Osteoarthr Cartil Open. 2024 Oct 9;6(4):100526. doi: 10.1016/j.ocarto.2024.100526 (PMC11550359; doi:10.1016/j.ocarto.2024.100526)
Supplement: Multimedia component 1 [file mmc1.docx]

**Supplemental Information:** Walking Recovers Cartilage Compressive Strain In Vivo

**
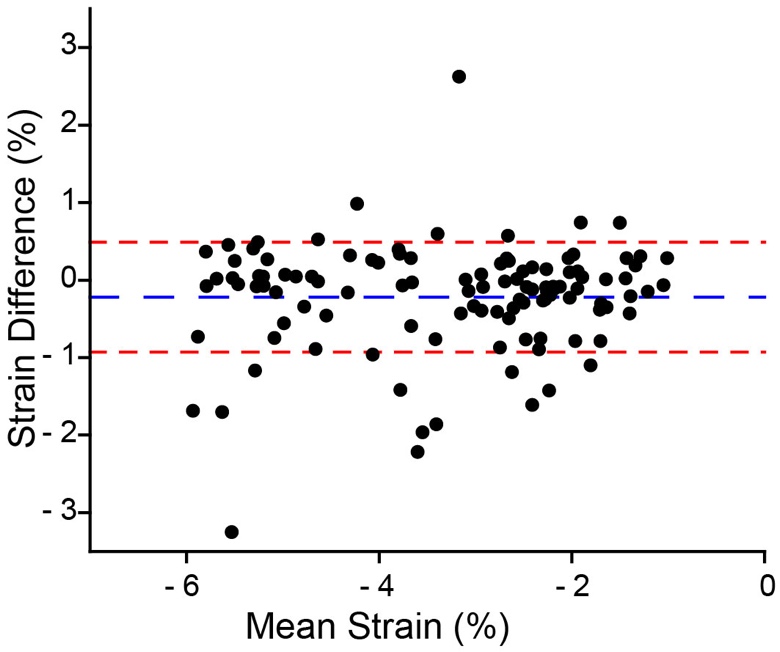
**

**Figure S1.** Intra-rater difference plot comparing the axial cartilage strain measured on the first and second pass. Slope and intercept are not significantly different than 0. The mean or bias (blue dashed line, -0.22% strain) is significantly different from zero based on a one sample t-test. Standard deviation (red dashed line) is ± 0.71% strain.


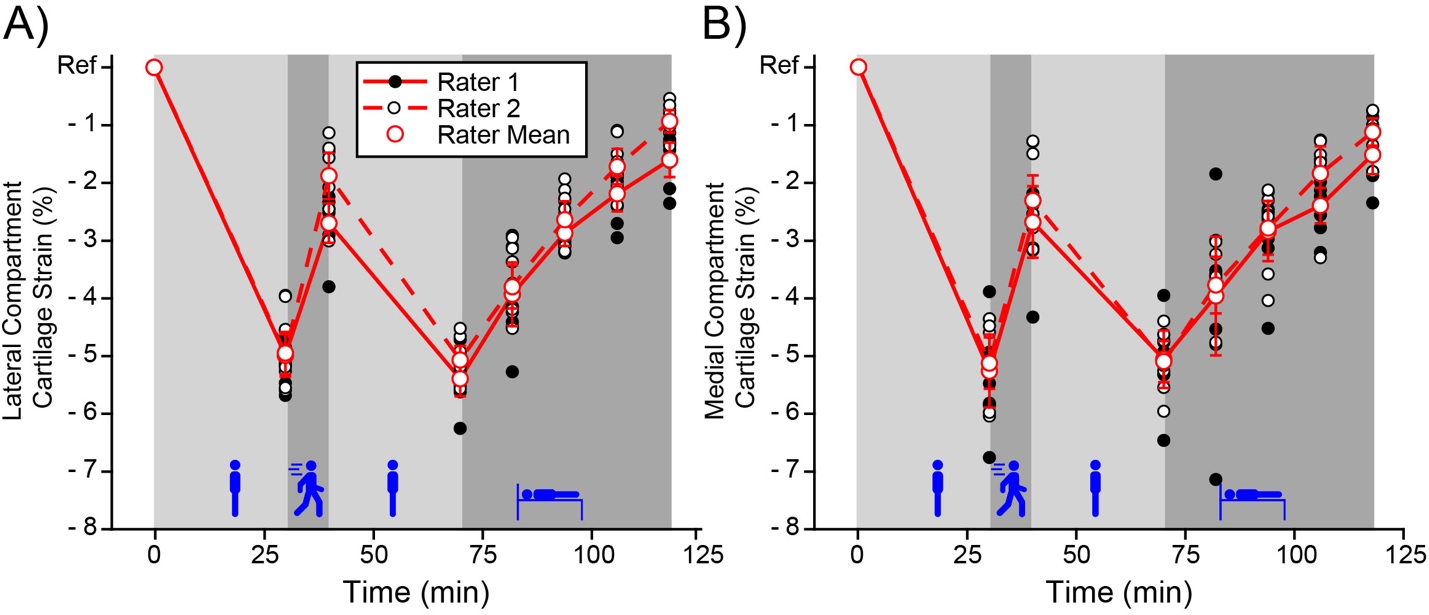


**Figure S2.** Inter-rater comparison for the (A) lateral and (B) medial compartments. The combined inter-rater bias (average difference) is -0.31% strain. The standard deviation of the differences is ± 0.94% strain.

**
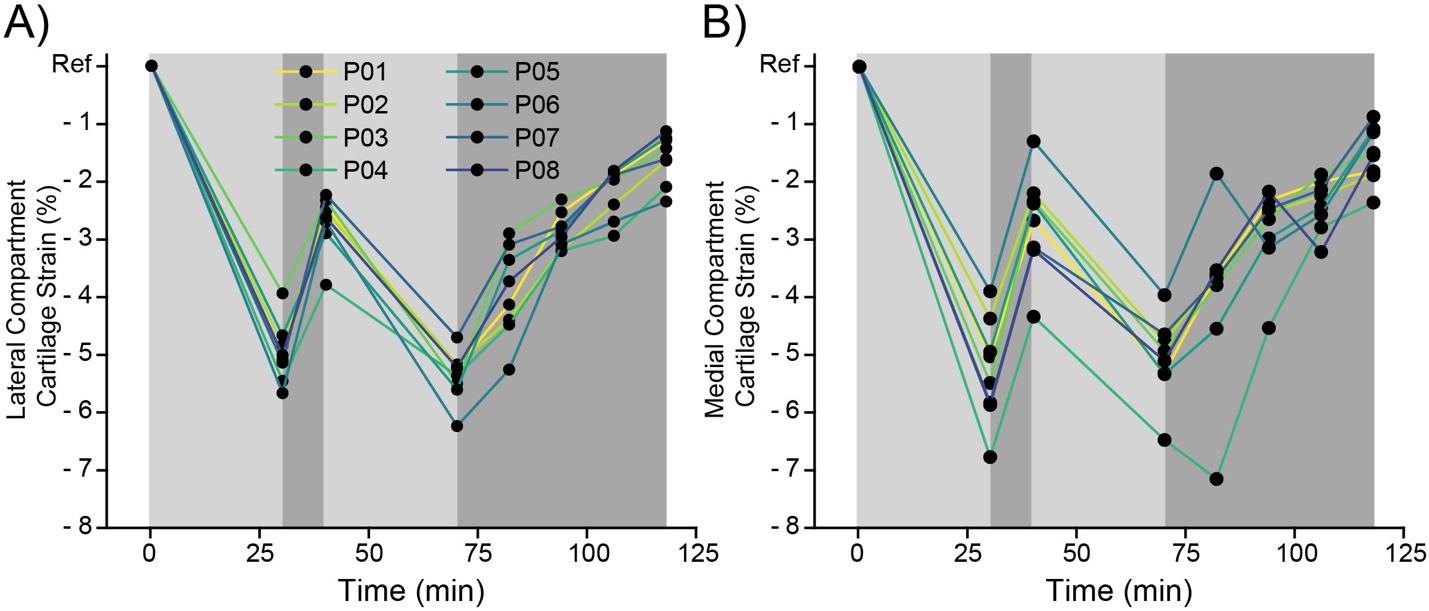
**

**Figure S3.** Connected lines for each participant (P01 to P08) in the (A) lateral and (B) medial compartments.
